# Global, regional, and national levels and trends in burden of urticaria: A systematic analysis for the Global Burden of Disease study 2019

**DOI:** 10.7189/jogh.14.04095

**Published:** 2024-05-31

**Authors:** Yuanchun Pu, Liyu He, Xiangyu Wang, Yaodong Zhang, Shidi Zhao, Jinhai Fan

**Affiliations:** 1 Department of Urology, The First Affiliated Hospital of Xi’an Jiaotong University, Xi’an, China; 2 Department of Radiology, The First Affiliated Hospital of Xi’an Jiaotong University, Xi’an, China; 3 Department of Urology, The Second Affiliated Hospital of Xi’an Jiaotong University (Xibei Hospital), Xi’an, China; 4 Cancer Center, The First Affiliated Hospital of Xi’an Jiaotong University, Xi’an, China

## Abstract

**Background:**

Urticaria places a significant burden on individuals and society due to its widespread nature. The aim of this study was to evaluate the burden of urticaria in different regions and nations by analysing data from the Global Burden of Disease study 2019 (GBD 2019), with the goal of providing information to health care policymakers.

**Methods:**

By utilising data from the GBD 2019 database, this study analysed metrics such as incidence, prevalence, disability-adjusted life years (DALYs), age-standardised rate (ASR), and estimated annual percentage changes (EAPC) globally and across 204 countries and regions. The data was further stratified by age, sex, and sociodemographic index (SDI).

**Results:**

In 2019, global incidence cases, prevalence cases, and overall disease burden as measured by DALYs all increased. The distribution of the burden exhibited marked geographical heterogeneity. At the regional level, the burden is highest in Central and Eastern Europe and Central Asia, with the strongest growth in South Asia, compared with a decline in the high-income Asia Pacific. At the country level, Nepal reports the highest burden of urticaria, while Portugal has the lowest. Gender and age analyses showed that the burden of urticaria is higher in females than in males, with urticaria cases declining with age, especially in children, and picking up among the elderly. The study also finds a correlation between the burden of urticaria and the SDI, with the central part of the SDI showing a consistent increasing trend.

**Conclusion:**

This study found that the global burden of urticaria has risen from 1990 to 2019. Factors like geographic location, gender, and SDI influenced the urticaria burden. Overall, these results offer a resource to guide public health strategies seeking to reduce the burden of urticaria.

Urticaria is a transient dermatological phenomenon with 160 million global incidence cases and 86 million global prevalence in 2017 [1]. And it was demonstrated that chronic urticaria alone possesses a lifetime prevalence rate of 1.4%, excluding other types of urticaria [2]. Symptomatically, urticaria is characterised by pruritic, erythematous, and elevated plaques, often resulting from the degranulation of mast cells in the superficial dermis releasing histamine, and usually resolves within 24 hours [3–5]. Another manifestation associated with urticaria is angioedema, involving deeper blood vessels, specifically small veins, and these two conditions can sometimes coexist [3,5]. These clinical symptoms of urticaria place a heavy burden of disease on patients. There are studies using the Chronic Urticaria Quality of Life Questionnaire (CU-Q2oL) and Dermatology Life Quality Index (DLQI) surveys which show that these symptoms of urticaria can have a significant impact on patients' sleep and daily life, as well as the work performance and productivity [6].

Beyond the symptoms, urticaria causes a burdensome public health burden. There also are some studies showing the incidence rates exhibit significant variance across different countries, which means that the burden of urticaria is common and variable [7–9]. The variability of burden is reflected in many ways, with the highest prevalence of acute urticaria (AU) in children. Women are more prone to urticaria, but with a smaller proportion of chronic urticaria (CU) [10]. Meanwhile, studies in several countries have reported a rising trend in the public health impact of urticaria and suggest that there is an urgent need to address barriers to treatment and reduce the burden [11–13]. Ultimately, urticaria leads to high social expenditures, both in terms of medical resources and financial resources [7,14].

Current research efforts are gradually shedding light on the multifaceted nature of urticaria, but gaps remain in the overall analysis of the global burden and the sociodemographic determinants that influence its burden and severity [1,11,12,15,16]. In response, our research delves into the incidence, prevalence, and disability-adjusted life years (DALYs) and analyses the estimated annual percentage change (EAPC) and age-adjusted percentage change (AAPC) to evaluate the burden of urticaria and explains its developing trends at global, regional, national. In particular, we use a composite indicator, the sociodemographic index (SDI), which facilitates the assessment of the impact of fertility rate, educational attainment, and economy on the burden of urticaria as well as on development [17]. Our ultimate goal is to increase policymakers' attention to the urticaria burden and promote rational allocation of health resources.

## METHODS

### Data source

The Global Burden of Disease (GBD) database is an open-access public database with data from literature, survey data, and claims data subjected to age-sex stratification. The GBD data of urticaria were analysed by the DisMod-MR 2.1 computational tool with the integration of study-level covariates (Claims data – 2000), aimed at getting the prevalence and incidence of urticaria, classified by location, year, age, and sex. At the same time, information collected from the Medical Expenditure Panel Survey was used for the meta-analysis of the proportion of urticaria cases classified as either mild or severe. These bifurcated data streams were then combined and allocated Disability Weights. By using the Comorbidity Corrections (COMO), the data can calculate the years lived with disability (YLDs), which were further used to estimate DALYs [18].

The most recent GBD 2019 data resources is used in this study and obtained by the Global Health Data Exchange (GHDx) query tool (http://ghdx.healthdata.org/gbd-results-tool, accessed on 26 September 2022) [19]. We selected the data conforming to ICD10 urticaria diagnostic criteria (ICD-10:50) with no additional inclusions or exclusions. Symptoms of urticaria are categorised into two levels, and both get the corresponding disability weights: mild urticaria (severity level 1, disability weight (DW) (95% confidence interval (95% CI) = 0.027 (0.015, 0.042))); severe urticaria (severity level 2, DW (95% CI = 0.188 (0.124, 0.267))) [18]. The data we obtained includes multiple epidemiological indicators of 23 age groups, including incidence, prevalence, and YLDs, DALYs, and metrics used for these indicators include number, rate, percent, and years. Gender is grouped into males, females, and both sexes. The geographical scope comprises 204 countries and regions, divided into 21 regions and seven larger areas (WHO regions, World Bank income levels, and more). Instead of using the human development index (HDI) in other studies, we chose the sociodemographic index (SDI) to make more accurate comparisons of the indicators related to medical health, which is the geometric mean of the mean education for those ages 15 and older (EDU15+), total fertility rate under the age of 25 (TFU25) and lag distributed income (LDI) per capita. Sociodemographic index was stratified into five different levels: low SDI (0, 0.454743), low-middle SDI (0.45474, 0.607679), middle SDI (0.607679, 0.689504), high-middle SDI (0.689504, 0.805129), and high SDI (0.805129, 1) [17].

### Statistical analysis

In this study, we calculate age-standardised rate (ASR), EAPC, and AAPC. Age-standardised rat is used to neutralise the influence of age-structural differences when comparing urticaria risks. The calculation formula is:



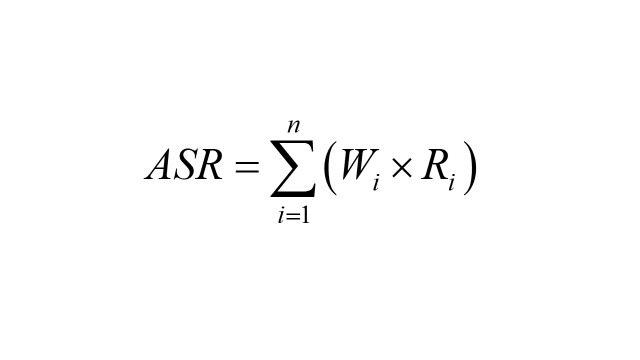



(*W_i_* is the proportion of the *i^th^* age group in the standard population; *R_i_* is the crude rate for the *i^th^* age group in the observed population; *n* is the number of age group). Estimated annual percentage changes is used to describe the trend of rate over a time period, and it’s calculated via a log-linear model: ln(*y*) = α + β × calendar year + ϵ (*y* is the annual rate; τshows the positive or negative trends of ASR; ϵ is the random error term) and EAPC is calculated as:



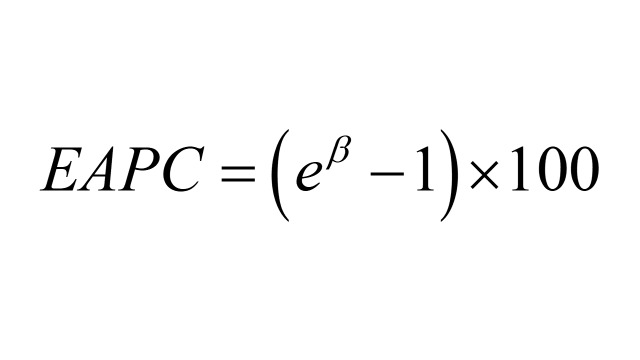
.

Like EAPC, AAPC is also used to assess long-term trends but represents a weighted average of EAPCs over a given time span. It is estimated through the Joinpoint model:



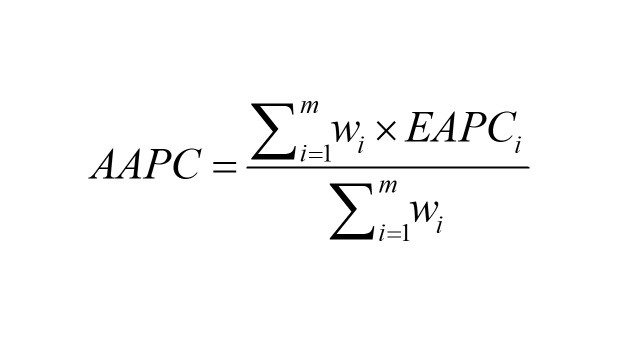



(*EAPC_i_* is the EAPC in the *i^th^* time; *W_i_* is the length of the *i^th^* time; *m* is the number of time segments). The criterion for statistical significance in our study was a *P*-value (*p* in figures) of less than 0.05. To address the increased risk of Type I error associated with multiple hypotheses, we used Benjamini and Hochberg False Discovery Rate (FDR) correction to adjust the *P*-value and improve the reliability of the results.

Data analysis and data visualisation were executed via R software (Version 4.1.2, 2021, Vienna, Austria, https://www.r-project.org) and Joinpoint Trend Analysis Software (Versions 4.9.0.1, 2022, Statistical Methodology and Applications Branch, Surveillance Research Program, National Cancer Institute, https://surveillance.cancer.gov/joinpoint/).

## RESULTS

### Global urticaria burden

**Figure 1:**
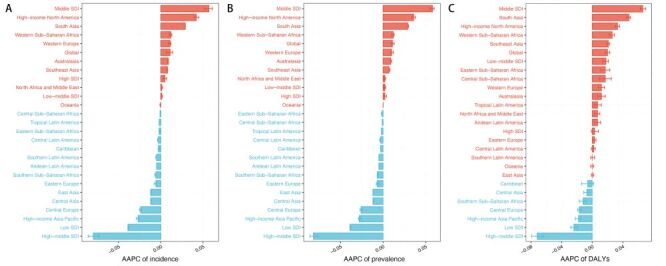
Age-adjusted percentage chang (AAPC) of the age-standardised incidence, age-standardised prevalence, age-standardised disability-adjusted life years (DALYs) for urticaria from 1990 to 2019. Red represents values greater than 0 and blue represents values less than 0. **Panel A.** AAPC of incidence. **Panel B.** AAPC of prevalence. **Panel C.** AAPC of DALYs.

In 2019, there were 114.709 million (95% uncertainty interval (UI) = 101.310, 129.286) incidence cases globally with an age-standardised incidence rate (ASIR) equating to 1527.5 (95% UI = 1346.2, 1726.5) per 100 000 population. Meanwhile, the EAPC (0.0220 (95% UI = 0.0176, 0.0263)) and AAPC of incidence were both noted to be greater than 0, thereby showing an upward trend in the ASIR from the period of 1990 to 2019 (**Figure 1**, panel A, Table S1 in the **Online Supplementary Document**).

Simultaneously, there were 65.140 million (95% UI = 57.517, 73.499) prevalence cases globally in 2019, with an age-standardised prevalence rate (ASPR) equating to 865.5 (95% UI = 761.8, 980.6) per 100 000 population. The EAPC (0.0234 (95% UI = 0.0192, 0.0276)) and AAPC of prevalence both exceeded 0, thereby showing an upward trend in the ASPR spanning from 1990 to 2019 (**Table 1****,** **Figure 1**, panel B).

**Table 1:** The prevalence cases and ASR per 100 000 of urticaria in 1990 and 2019, and the EAPC of prevalence spanning 1990–2019

	1990		2019		1990-2019
	**Prevalence cases**	**ASR per 100 000**	**Prevalence cases**	**ASR per 100 000**	**EAPC**
	**No. (95% UI), in millions**	**No. (95% UI)**	**No. (95% UI), in millions**	**No. (95% UI)**	**No. (95% UI)**
Global	48.088 (41.843, 54.883)	862.6 (759.5, 975.8)	65.140 (57.517, 73.499)	865.5 (761.8, 980.6)	0.0234 (0.0192, 0.0276)
Sex					
*Male*	19.654 (17.068, 22.549)	692.4 (607.8, 784.9)	26.783 (23.596, 30.261)	705.5 (619.1, 798.8)	0.0780 (0.0733, 0.0826)
*Female*	28.435 (24.747, 32.412)	1035.1 (909.5, 1173.5)	38.357 (33.958, 43.336)	1028.9 (903.9, 1167.3)	−0.0117 (−0.0156, −0.0077)
Social-demographic index					
*Low SDI*	5.569 (4.744, 6.541)	902.2 (791.9, 1028.3)	11.276 (9.631, 13.228)	892.1 (781.0, 1018.5)	−0.0372 (−0.0383, −0.0362)
*Low-middle SDI*	11.519 (9.821, 13.496)	914.0 (797.8, 1044.4)	16.318 (14.178, 18.706)	914.5 (798.3, 1045.9)	0.0099 (0.0071, 0.0128)
*Middle SDI*	14.861 (12.846, 17.124)	820.6 (718.2, 930.2)	19.162 (16.870, 21.554)	834.6 (731.3, 946.7)	0.0741 (0.0691, 0.0791)
*High-middle SDI*	9.890 (8.688, 11.195)	869.9 (762.3, 985.8)	11.006 (9.741, 12.294)	849.6 (744.5, 960.9)	−0.059 (−0.0756, −0.0423)
*High SDI*	6.226 (5.663, 6.805)	792.6 (719.0, 872.0)	7.344 (6.742, 7.967)	793.4 (722.9, 868.7)	−0.0014 (−0.0098, 0.0070)
Region					
*Central Asia*	0.846 (0.719, 0.988)	1101.0 (952.5, 1259.8)	1.033 (0.892, 1.189)	1097.3 (949.1, 1255.3)	−0.0113 (−0.012, −0.0107)
*Central Europe*	1.364 (1.210, 1.534)	1198.2 (1049.5, 1364.1)	1.131 (1.024, 1.249)	1189.3 (1054.2, 1337.9)	−0.013 (−0.0187, −0.0074)
*Eastern Europe*	2.449 (2.148, 2.778)	1162.6 (1007.4 - 1340.1)	2.114 (1.875, 2.377)	1160.3 (1005.3, 1337.0)	−0.0035 (−0.0047, −0.0023)
*Australasia*	0.180 (0.158, 0.203)	910.3 (798.1, 1030.6)	0.249 (0.221, 0.280)	912.7 (800.5, 1034.4)	0.0069 (0.0052, 0.0086)
*High-income Asia Pacific*	1.283 (1.131, 1.445)	791.8 (694.2, 902.9)	1.260 (1.120, 1.403)	785.5 (688.1, 896.9)	−0.0249 (−0.0264, −0.0235)
*High-income North America*	2.349 (2.204, 2.496)	885.4 (829.4, 946.3)	2.906 (2.749, 3.069)	895.5 (841.3, 955.0)	0.0075 (−0.0038, 0.0187)
*Southern Latin America*	0.383 (0.334, 0.437)	763.7 (668.1, 868.7)	0.484 (0.426, 0.547)	762.7 (667.1, 867.6)	−0.0045 (−0.005, −0.0040)
*Western Europe*	2.325 (2.079, 2.566)	586.2 (521.4, 652.1)	2.654 (2.384, 2.924)	588.0 (522.9, 653.8)	0.0112 (0.0091, 0.0132)
*Andean Latin America*	0.336 (0.287, 0.392)	800.8 (699.1, 917.8)	0.500 (0.444, 0.584)	799.6 (698.1, 916.3)	−0.0069 (−0.0074, −0.0063)
*Caribbean*	0.294 (0.254, 0.340)	801.4 (699.6, 918.5)	0.365 (0.320, 0.414)	800.8 (699.1, 917.7)	−0.0035 (−0.004, −0.0031)
*Central Latin America*	1.484 (1.273, 1.734)	826.0 (723.6, 945.8)	2.019 (1.768, 2.298)	825.1 (722.9, 944.6)	−0.0044 (−0.0048, −0.0040)
*Tropical Latin America*	1.368 (1.179, 1.586)	847.2 (741.4, 969.0)	1.790 (1.574, 2.023)	846.7 (741.0, 968.2)	−0.0024 (−0.0027, −0.0021)
*North Africa and Middle East*	3.654 (3.118, 4.235)	950.0 (829.0, 1075.6)	5.833 (5.071, 6.636)	950.5 (828.9, 1075.5)	0.0042 (0.0022, 0.0062)
*South Asia*	12.115 (10.392, 14.214)	981.1 (856.2, 1122.7)	17.852 (15.541, 20.451)	989.5 (863.2, 1133.9)	0.0309 (0.0291, 0.0328)
*East Asia*	9.284 (8.037, 10.639)	740.1 (646.6, 841.8)	9.960 (8.771, 11.132)	737.5 (644.6, 838.5)	−0.0105 (−0.0113, −0.0097)
*Oceania*	0.049 (0.042, 0.057)	694.0 (609.9, 791.1)	0.097 (0.084, 0.112)	694.0 (609.9, 791.0)	−0.0012 (−0.0016, −0.0008)
*Southeast Asia*	3.648 (3.145, 4.208)	740.2 (647.7, 841.3)	4.944 (4.322, 5.612)	741.7 (648.8, 843.0)	−0.0045 (−0.005, −0.004)
*Central Sub-Saharan Africa*	0.523 (0.444,0.620)	802.2 (700.3, 919.3)	1.196 (1.017, 1.409)	802.0 (700.2, 918.8)	−0.0008 (−0.0015, −0.0001)
*Eastern Sub-Saharan Africa*	1.834 (1.553, 2.176)	819.7 (717.8, 938.4)	3.809 (3.245, 4.491)	819.4 (717.7, 938.1)	−0.0028 (−0.0034, −0.0023)
*Southern Sub-Saharan Africa*	0.479 (0.411, 0.559)	837.4 (732.8, 957.3)	0.667 (0.581, 0.767)	835.8 (731.3, 954.9)	−0.0072 (−0.0106, −0.0037)
*Western Sub-Saharan Africa*	1.844 (1.564, 2.181)	822.5 (720.5, 941.4)	4.269 (3.638, 5.038)	825.3 (723.0, 944.5)	0.0137 (0.0099, 0.0175)

ASR – age-standardised rate, EAPC – estimated annual percentage change, UI – uncertainty interval

Regarding DALYs, the global burden attributable to urticaria measured 3.899 million (95% UI = 2.554, 5.584), and age-standardised of DALYs measured 51.9 (95% UI = 34.0, 75.1) per 100 000 population, and both EAPC (0.0338 (95% UI = 0.0287, 0.0388)) and AAPC of DALYs were both greater than 0, thereby showing an upward trend from 1990 to 2019 (**Figure 1**, panel C, Table S2 in the Online **Supplementary Document**).

### Regional urticaria burden

With respect to the ASIR, the highest rate in 2019 appeared in Central Europe (2097.6 (95% UI = 1862.6, 2363.4)), Eastern Europe (2043.6 (95% UI = 1771.5, 2355.2)), Central Asia (1932.4 (95% UI = 1678.2, 2216.4)), with the lowest rate in East Asia (1303.6 (95% UI = 1143.2, 1488.5)), Oceania (1226.7 (95% UI = 1079.6, 1402.7)), Western Europe (1040.2 (95% UI = 927.3, 1155.9)). Concerning the EAPC of incidence, the strongest upward trend was identified in South Asia (0.0307 (95% UI = 0.0289, 0.0325)), while the strongest downward trend region is high-income Asia Pacific (−0.0242 (95% UI = −0.0258, −0.0227)) (Table S1 in the **Online Supplementary Document**).

In the ASPR part, the apogee was observed in Central Europe (1189.3 (95% UI = 1054.2, 1337.9)), Eastern Europe (1160.3 (95% UI = 1005.3, 1337.0)), and Central Asia (1097.3 (95% UI = 949.1, 1255.3)), with the lowest rate in East Asia (737.5 (95% UI = 644.6, 838.5)), Oceania (694.0 (95% UI = 609.9, 791.0)) and Western Europe (588.0 (95% UI = 522.9, 653.8)). Concerning the EAPC of prevalence, the strongest upward trend was identified in South Asia (0.0309 (95% UI = 0.0291, 0.0328)), while the strongest downward trend region is high-income Asia Pacific (−0.0249 (95% UI = −0.0264, −0.0235)) (**Table 1**).

In the age-standardised of DALYs part, the apogee was observed in Central Europe (71.9 (95% UI = 47.2, 103.4)), Eastern Europe (70.1 (95% UI = 46.0, 102.1)), and Central Asia (66.2 (95% UI = 43.3, 96.0)), with the lowest rate in Western Europe (34.9 (95% UI = 23.0, 49.4)), Oceania (41.4 (95% UI = 27.1, 59.8)), and East Asia (44.6 (95% UI = 29.1, 64.8)). Concerning the EAPC of DALYs, the strongest upward trend region was identified in South Asia (0.0518 (95% UI = 0.0497, 0.0540)), while the strongest downward trend region is high-income Asia Pacific (−0.0149 (95% UI = −0.0172, −0.0126)) (Table S2 in the **Online Supplementary Document**).

For the regional AAPC of the incidence, high-income North America exhibited the biggest upward trend, succeeded by South Asia, which also manifested an increasing trend. Conversely, Central Europe and high-income Asia Pacific exhibited the most significant downward trends. Similarly, the AAPC of the prevalence showed the biggest increasing trend in high-income North America and succeeded by South Asia, while Central Europe and high-income Asia Pacific exhibited the biggest decreasing trend. For the regional AAPC of DALYs, South Asia exhibited the biggest increasing trend and succeeded by high-income North America, while Central Europe and high-income Asia Pacific exhibited the biggest decreasing trend (**Figure 1**, panels A–C).

### National urticaria burden

**Figure 2:**
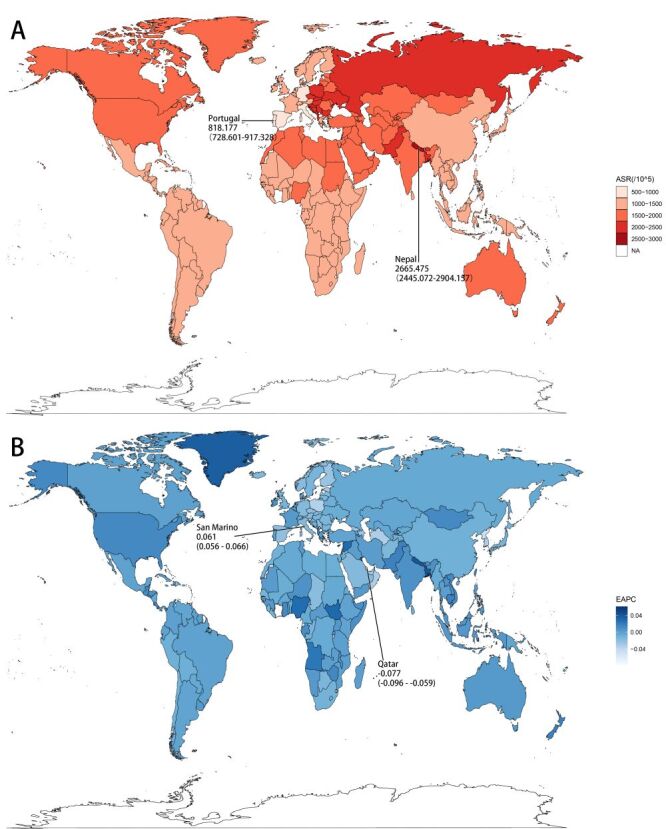
The urticaria global age-standardised rate (ASR) (per 10^5) incidence map and estimated annual percentage changes (EAPC) of incidence map in 2019 by countries and territories. **Panel A.** ASR (per 10^5) incidence map. **Panel B.** EAPC of incidence map.

At the national scale, the data for 2019 exhibit Nepal got the highest ASIR, ASPR, and the age-standardised of DALYs at the same time. Conversely, Portugal exhibited the lowest figures in these key indicators (**Figure 2**, panel A, Figures S1A–2A in the **Online Supplementary Document****)**. The EAPC of the incidence has the biggest upward trend in San Marino, Andorra, Nepal, Bangladesh, and Greenland and the biggest decreasing trend in Qatar, Equatorial Guinea, Democratic People's Republic of Korea, Malta, and Cabo Verde (**Figure 2**, panel B). This sequence similarly applies to the EAPC of prevalence and DALYs (Figures S1B–2B in the **Online Supplementary Document**). In addition, we retained small countries or regions with populations of less than 1 000 000 for the objectivity of data, such as San Marino, Andorra, Greenland, Malta, Cape Verde, etc., but their accuracy and extensiveness are not clear.

### Age and sex

The data indicate a decline in urticaria incidence and prevalence concomitant with age growth, especially within the 1–14 years old. Thereafter, the decline tended to weaken gradually, yet a slight resurgence is observed between 50 and 69 years old. Gender-specific analysis shows that EAPCs for incidence, prevalence, and DALYs for females are negative values, meaning a declining trend, while for males are positive values, indicating an upward trend (**Table 1**, Tables S1–2, Figures S3–5 in the **Online Supplementary Document**).

### Sociodemographic index

**Figure 3:**
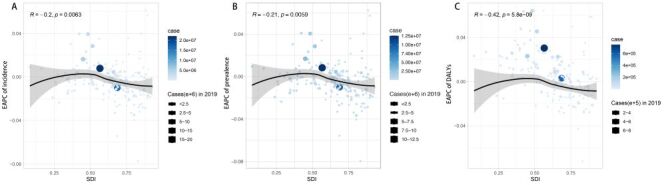
The correlation between urticaria estimated annual percentage changes (EAPC) and sociodemographic index (SDI) levels in 2019. **Panel A.** EAPC of age-standardised incidence rate (ASIR) and SDI correlation. **Panel B.** EAPC of age-standardised prevalence rate (ASPR) and SDI correlation. **Panel C.** EAPC of disability-adjusted life years (DALYs) and SDI correlation.

In the correlation analysis between incidence EAPC and SDI, there was a mildly negative correlation (*R* = −0.2, *P* = 0.0063). Since the *R*-value (Pearson correlation coefficient) is not very close to −1, this correlation is not completely linear. Most of the low-middle SDI level corresponded to positive values for the EAPC of incidence fitted curve, while all medium-high SDI and high SDI values corresponded to negative values (**Figure 3**, panel A). In the relationship between ASIR and regional SDI, there is a slightly positive correlation (*R* = 0.1, *P* = 0.0065). Specifically, regions such as Central Europe, Eastern Europe, Central Asia, and South Asia deviate above the fitted curve, indicating that their actual values surpass the expected. Conversely, regions like Western Europe, Oceania, East Asia, Southeast Asia, and Southern Latin America fall below the fitted curves, indicating that their actual values are less than projected (Figure S6A in the **Online Supplementary Document**). For ASIR correlated with country-level SDI, there was no linear obvious trend (*P* = 0.56) (Figure S6B in the **Online Supplementary Document**).

**Figure 4:**
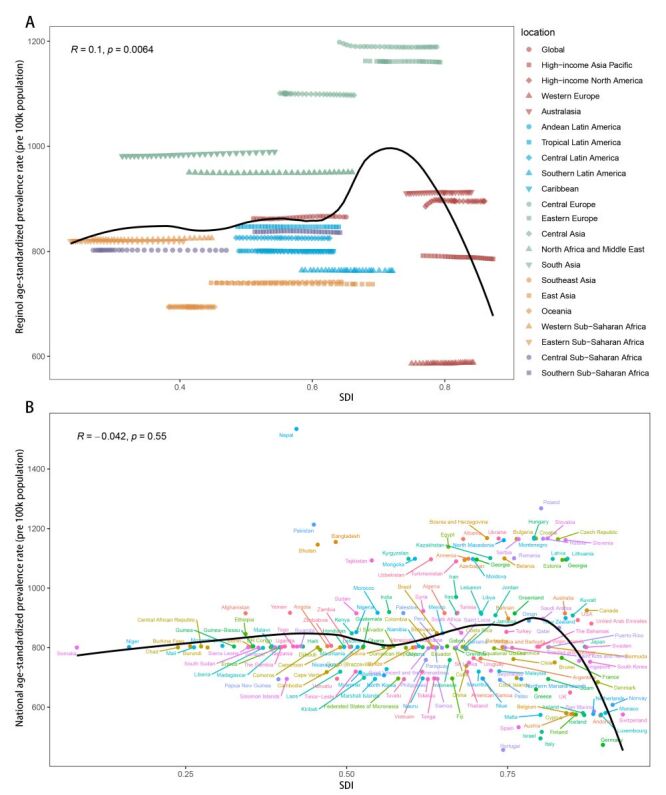
The correlation between urticaria age-standardised prevalence rate (ASPR) and sociodemographic index (SDI) levels in 2019. **Panel A.** Regional ASPR and SDI correlation from 1990 to 2019. **Panel B.** National ASPR and SDI correlation in 2019.

In the correlation analysis between the prevalence EAPC and SDI showed a mildly negative correlation (*R* = −0.21, *P* = 0.0059), which is not completely linear. And the distribution pattern of SDI levels corresponding to EAPC of prevalence values was similar to incidence (**Figure 3**, panel B). In the association between ASPR and regional SDI, there is a slightly positive correlation (*R* = 0.1, *P* = 0.0064). Similar to the incidence, Central Europe, Eastern Europe, Central Asia, and South Asia exceeded the fitted curve, while Western Europe, Oceania, East Asia, Southeast Asia, and Southern Latin America fell below the fitted curves (**Figure 4**, panel A). For ASPR and country SDI, there was no obvious linear trend (*P* = 0.55) (**Figure 4**, panel B).

Concerning the correlation between DALYs EAPC and SDI, they are moderately negatively correlated (R = −0.42, *P* = 5.8e-09), which is not completely linear. And the distribution pattern of SDI levels corresponding to EAPC of DALYs values was similar to incidence (**Figure 3**, panel C). In the relationship between age-standardised DALYs and regional SDI, there is a slightly positive correlation (*R* = 0.12, *P* = 0.0016). Regions such as Central Europe, Eastern Europe, and Central Asia exceeded the fitted curve, while Western Europe, Oceania, East Asia, Southeast Asia, and Southern Latin America fell below the fitted curves (Figure S7A in the **Online Supplementary Document**). For the age-standardised DALYs and country SDI, there was no obvious linear trend (*P* = 0.64) (Figure S7B in the **Online Supplementary Document**).

**Figure 5:**
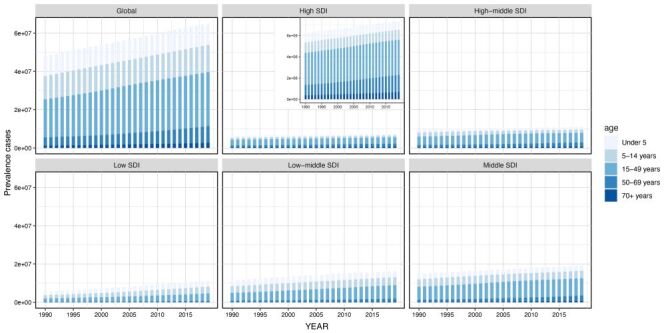
The urticaria prevalence cases of five age group in Global and 5SDI level from 1990 to 2019. SDI – sociodemographic index

Simultaneously, the incidence, prevalence, and DALYs cases in the low-middle and middle SDI were significantly higher than those in other SDI strata. The main cases across all SDI categories were concentrated in the 15–49 age bracket, albeit the prevalence of cases under age 14 was higher in low, low-middle, and middle SDI compared to high-middle and high SDI categories (**Figure 5****,** Figures S8–9 in the **Online Supplementary Document**).

In the Joinpoint analysis spanning from 1990 to 2019, both ASIR, ASPR, and DALYs in low SDI and high-middle SDI categories displayed a continuous decline, whereas a consistent increase was observed in middle SDI. By contrast, the remaining SDI categories showed fluctuating trends. When looking only at 2019, all SDI regional burden indicators decline, except for the middle SDI, which remains flat (Figures S10–12 in the **Online Supplementary Document**). Age-adjusted percentage change, derived from Joinpoint analysis, showed that middle SDI got the highest among all AAPC values for incidence, prevalence, and DALYs, while the lowest was noted in high-middle and low SDI (**Figure 1**, panels A–C).

## DISCUSSION

Using the urticaria data set from the GBD study in 2019, this study is dedicated to discovering changes in the burden of urticaria, its distributional characteristics, and associations with development index. Based on this purpose of the study, we found that the global burden of urticaria showed an increasing trend, and the burden of urticaria showed significant geographic and gender heterogeneity. In particular, SDI correlated with multiple aspects of the burden of urticaria.

The Global urticaria metrics, including ASIR, ASPR, and age-standardised DALYs for urticaria, show an increased trend, which is basically similar to the results of previous studies. It can be determined what may cause the burden to rise based on the available research. There are observational studies have found an increase in allergic diseases due to increased temperatures. Simultaneously, there are observational studies indicating that the rise in temperature has led to an increase of allergic diseases [20,21]. This change may be due to the alteration in antigen exposure and the decrease in the immune system's tolerance to antigen specificity caused by the rising temperatures [22]. However, attributing the increased burden directly to climate change is extremely difficult to quantify. Differing from most diseases, urticaria can recur within a single year, increasing its incidence, as each recurrence is counted as a new case [23,24].

Different countries and regions show geographical heterogeneity, which may be due to environmental and social factors. At the regional level of ASIR, ASPR, and age-standardised DALYs, Central Europe, Eastern Europe, and Central Asia exhibit the highest values, whereas East Asia, Oceania, and Western Europe register the lowest. The probable reason for this is that Central Europe, Eastern Europe, and Central Asia are all inland areas, in contrast to East Asia, Oceania, and Western Europe being near the ocean. This observation is consistent with existing time-series experiments indicating that increase of relative humidity can reduce the number of urticaria outpatient visits [25,26]. Meanwhile, multiple observational studies have confirmed that exposure to air pollutants such as O_3_, PM_2.5_, and PM_10_ increases the risk of urticaria and exhibits a cumulative effect [20,26]. We find that South Asia has the largest growth trends in ASIR, ASPR, and DALYs. This trend may be due to the rapid economic development represented by countries like India and Bangladesh. Such development is accompanied by swift population growth and advances in local medical technology, resulting in increased outpatient visits. Moreover, industrial growth contributes to pollution, thereby exacerbating both the incidence and prevalence of diseases in the South Asia region [27]. At the national level, Nepal obtains the highest values in ASIR, ASPR, and age-standardised DALYs. We believe that the factors for such a phenomenon are multifactorial. Geographically located in high-altitude areas are subjected to strong UV rays, which are more likely to damage the skin barrier, consequently instigating the incidence of urticaria [13,28]. Concurrently, the insect and pollen of abundant vegetation in the southern Himalayan are common allergens for urticaria [29]. At the same time, Nepal, as a South Asian country, also applies to the analysis of South Asian countries above.

In terms of gender heterogeneity, the burden of females is higher than that of males. In all age groups, females exhibit higher values of ASIR, ASPR, and age-standardised DALYs than males, resembling previous research findings [1,2,6,9,16]. The high ASIR and ASPR in females are speculated to be influenced by oestrogen, as animal studies have proved its capacity to promote mast cell activation and allergic sensitisation [30]. Additionally, research shows that air pollution is more likely to harm female skin [31]. In terms of age, the incidence, prevalence, and DALYs for the <14 years age group significantly outweigh those for the >14 years age group, confirming prior studies as well [1,26,32]. But we found a rising trend of anomalies at ages 50-60 that was opposite to the overall trend, especially in females. Not only us but also other studies have observed a similar phenomenon, with the main peak of prevalence distributed between ages 30–59 [11,12,33]. Several studies have found that the prevalence of chronic spontaneous urticaria (CSU) is higher in the elderly compared to the young. Also, comorbidities and multiple organ dysfunction result in more severe and prolonged symptoms, which are more likely to result in medical attention and to be documented by health care or insurance claims systems [34–36].

In the correlation analysis between the SDI and the burden of urticaria, the Pearson value suggests that SDI shows a slightly negative linear correlation with EAPC of incidence, EAPC of prevalence, and a moderate negative linear correlation with EAPC of DALYs in 2019. From the composition of SDI indicators, SDI was positively correlated with EDU15+ and LDI, and negatively correlated with TFU25 [37]. Therefore, we hypothesise that improvements in health and education are negatively correlated with the EAPC of burden, which means that the burden of disease is decreasing or growing at a slower rate. At the regional level, SDI shows a slightly positive correlation with the ASR burden. There are studies similar to ours indicate that children from households with higher incomes face higher risks of urticaria compared to children from lower-income households [38]. But the SDI does not have a linear correlation with the ASIR, ASPR, and ASR of DALYs at the national level. We consider that such results are due to nonlinear correlations or the influence of confounding variables other than SDI. Most obviously, the middle SDI groups show the biggest AAPC, the highest burden in 5SDI statistics of 2019, and the most significant burden increase trend in Joinpoint graphs from 1990–2019. The special features of middle SDI groups are posited to be contributory factors. They are likely undergoing processes of industrialisation and urbanisation, increasing environmental and lifestyle-related disease triggers such as new environmental allergens, medications, and food additives, which leads to an increase in both the incidence and prevalence of urticaria [20,39]. At the same time, middle SDI regions may be facing additional socio-economic pressures, such as job stress and accelerated life rhythms, which could potentially lead to the release of neurotrophic factors, thereby promoting the activation of mast cells in the skin and resulting in the onset of urticaria [40–42].

This study represents a ground-breaking effort in which, through the analysis of GBD 2019, we identified trends in the increasing global impact of urticaria. This finding continues previous research and emphasises the growing public health importance of this disease. Uniquely, this study not only tracks the epidemiologic change of urticaria over time, but also reveals the complexity of its geographic and gender differences. Perhaps most notably, our study breaks new ground by establishing a correlation between SDI and multiple aspects of the burden of urticaria. This innovative association not only enriches the existing body of knowledge, but also paves the way for targeted public health interventions and policies.

This study still has some limitations. First, data quality is a significant concern in urticaria research. The insurance claims data often used for studies of urticaria exclude cases that would not result in a hospital visit, thus potentially underestimating the true prevalence. Additionally, using ICD-10 codes without distinguishing between different urticaria subtypes exacerbates the data problem while making it impossible to study different types. Second, the results of this survey may be affected by sampling bias, especially due to the lack of data from different demographic and geographic backgrounds (especially low-income countries). There were also differences in diagnostic expertise provided by health care providers, and the majority of urticaria diagnoses came from emergency doctors rather than dermatologists, which introduced bias. Third, the study was also challenged by some methodological limitations. Temporal changes in urticaria prevalence and diagnostic criteria, coupled with advances in medical knowledge and technology, create difficulties in accurately attributing changes in burden to specific causes or interventions. The failure to distinguish between subtypes of urticaria further complicates the understanding of its prevalence and burden. Additionally, differences in data collection methods and heterogeneity in study designs, such as using self-report questionnaires, physician diagnoses, or insurance claims data, can lead to different results. In conclusion, future studies should aim to incorporate more refined, validated data sources, ensure the inclusion of diverse populations, and use standardised, rigorous methods. By addressing these issues, urticaria research can achieve greater accuracy and relevance, paving the way for improved public health strategies and interventions.

## CONCLUSIONS

This study indicates that the global burden of urticaria has risen significantly from 1990 to 2019, with clear geographical heterogeneity observed. These underscore the need for region-specific public health interventions. In addition, we found that the disease burden is generally higher among females than males. A correlation was also observed between the SDI and urticaria burden. These findings pinpoint the importance of additional epidemiological investigations and tailored policy efforts. More research is required to better understand etiological factors and most effectively guide strategies to relieve the increasing burden of urticaria.

## Additional material


Online Supplementary Document

**Correspondence to:** Jinhai Fan Department of Urology, The First Affiliated Hospital of Xi’an Jiaotong University 277 West Yanta Road, Xi’an People's Republic of China jinhaif029@126.com
