# Supplementary material for: Global, regional, and national levels and trends in burden of urticaria: A systematic analysis for the Global Burden of Disease study 2019
Source: J Glob Health. 2024 May 31;14:04095. doi: 10.7189/jogh.14.04095 (PMC11140429; doi:10.7189/jogh.14.04095)

Supplementary Material

This document contains supplementary information for the manuscript "Global, regional, and national levels and trends in burden of urticaria: a systematic analysis for the global burden of disease 2019 study".

Table S1. The incidence cases and ASR per 100,000 of urticaria in 1990 and 2019, and the EAPC of incidence spanning 1990-2019

	1990		2019		1990-2019
	incidence cases No. (95% UI) (millions)	ASR per 100,000 No. (95% UI)	incidence cases, No. (95% UI), (millions)	ASR per 100,000 No. (95% UI)	EAPC No. (95% UI)
Global	85.123 (74.625 - 96.974)	1522.7 (1343.0 - 1721.8)	114.709 (101.310 - 129.286)	1527.5 (1346.2 - 1726.5)	0.0220 (0.0176 - 0.0263)
Sex					
Male	34.804 (30.296 - 39.857)	1222.5 (1076.3 - 1387.7)	47.182 (41.660 - 53.423)	1245.3 (1096.8 - 1413.8)	0.0772 (0.0723 - 0.0821)
Female	50.319 (44.158 - 57.244)	1827.2 (1611.1 - 2065.9)	67.527 (59.700 - 75.961)	1815.9 (1601.5 - 2055.5)	-0.0137 (-0.0177 - -0.0097)
Social-demographic index					
Low SDI	9.965 (8.495 - 11.692)	1590.4 (1401.3 - 1801.3)	19.997 (17.123 - 23.308)	1572.5 (1384.6 - 1782.1)	-0.0373 (-0.0382 - -0.0364)
Low-middle SDI	20.475 (17.521 - 23.811)	1613.4 (1413.4 - 1838.7)	28.743 (25.132 - 32.899)	1613.9 (1413.8 - 1838.8)	0.0088 (0.0059 - 0.0116)
Middle SDI	26.280 (22.859 - 30.327)	1448.9 (1273.0 - 1645.7)	33.69 (29.634 - 38.029)	1473.0 (1294.6 - 1671.1)	0.0725 (0.0675 - 0.0775)
High-middle SDI	17.407 (15.303 - 19.716)	1534.3 (1346.7 - 1735.5)	19.322 (17.180 - 21.507)	1499.5 (1317.8 - 1696.8)	-0.0534 (-0.0693 - -0.0376)
High SDI	10.956 (9.951 - 12.006)	1399.7 (1269.5 - 1542.4)	12.895 (11.796 - 14.024)	1402.0 (1275.9 - 1540.0)	-0.0004 (-0.0091 - 0.0083)
Region					
Central Asia	1.495 (1.279 - 1.747)	1938.9 (1683.7 - 2223.4)	1.817 (1.570 - 2.089)	1932.4 (1678.2 - 2216.4)	-0.0113 (-0.012 - -0.0106)
Central Europe	2.388 (2.120 - 2.686)	2112.4 (1849.1 - 2403.1)	1.983 (1.796 - 2.191)	2097.6 (1862.6 - 2363.4)	-0.0122 (-0.0177 - -0.0068)
Eastern Europe	4.284 (3.757 - 4.868)	2047.5 (1775.2 - 2358.2)	3.703 (3.288 - 4.159)	2043.6 (1771.5 - 2355.2)	-0.0032 (-0.0044 - -0.0020)
Australasia	0.315 (0.279 - 0.355)	1600.3 (1407.9 - 1818.3)	0.435 (0.388 - 0.489)	1604.4 (1411.7 - 1823.9)	0.0065 (0.0048 - 0.0082)
High-income Asia Pacific	2.246 (1.987 - 2.540)	1397.0 (1229.6 - 1593.9)	2.204 (1.973 - 2.446)	1386.1 (1221.1 - 1582.1)	-0.0242 (-0.0258 - -0.0227)
High-income North America	4.146 (3.876 - 4.426)	1565.1 (1455.3 - 1687.5)	5.119 (4.812 - 5.442)	1585.9 (1477.3 - 1705.0)	0.0108 (-0.0018 - 0.0233)
Southern Latin America	0.675 (0.593 - 0.769)	1347.3 (1187.2 - 1532.9)	0.850 (0.752 - 0.955)	1345.3 (1185.6 - 1530.6)	-0.0046 (-0.0051 - -0.0041)
Western Europe	4.095 (3.669 - 4.533)	1036.9 (924.9 - 1152.7)	4.661 (4.201 - 5.158)	1040.2 (927.3 - 1155.9)	0.0115 (0.0094 - 0.0137)
Andean Latin America	0.596 (0.511 - 0.695)	1413.1 (1241.4 - 1605.5)	0.900 (0.788 - 1.024)	1411.1 (1239.6 - 1602.6)	-0.0066 (-0.0072 - -0.0061)
Caribbean	0.520 (0.453 - 0.598)	1414.2 (1242.4 - 1606.9)	0.643 (0.568 - 0.726)	1413.1 (1241.4 - 1605.0)	-0.0034 (-0.0039 - -0.0030)
Central Latin America	2.630 (2.261 - 3.070)	1457.7 (1278.3 - 1658.1)	3.554 (3.114 - 4.037)	1456.2 (1277.5 - 1656.1)	-0.0044 (-0.0048 - -0.0040)
Tropical Latin America	2.410 (2.081 - 2.784)	1495.2 (1305.8 - 1706.1)	3.145 (2.769 - 3.558)	1494.3 (1305.4 - 1704.9)	-0.0024 (-0.0027 - -0.0021)
North Africa and Middle East	6.480 (5.554 - 7.508)	1673.1 (1467.4 - 1903.5)	10.258 (8.983 - 11.722)	1673.5 (1467.4 - 1901.2)	0.0037 (0.0018 - 0.0056)

South Asia	21.510 (18.467 - 25.046)	1731.7 (1521.6 - 1977.9)	31.400 (27.475 - 35.943)	1746.4 (1533.8 - 1994.0)	0.0307 (0.0289 - 0.0325)
East Asia	16.385 (14.241 - 18.877)	1308.2 (1147.4 - 1493.8)	17.499 (15.478 - 19.511)	1303.6 (1143.2 - 1488.5)	-0.0105 (-0.0114 - -0.0096)
Oceania	0.087 (0.074 - 0.101)	1226.8 (1079.6 - 1402.9)	0.173 (0.150 - 0.201)	1226.7 (1079.6 - 1402.7)	-0.0013 (-0.0017 - -0.0009)
Southeast Asia	6.475 (5.604 - 7.524)	1309.4 (1148.8 - 1490.0)	8.716 (7.637 - 9.879)	1312.3 (1151.9 - 1492.8)	0.0077 (0.0073 - 0.008)
Central Sub-Saharan Africa	0.940 (0.795 - 1.114)	1415.5 (1243.8 - 1607.7)	2.122 (1.809 - 2.497)	1415.3 (1243.5 - 1607.4)	-0.0008 (-0.0014 - -0.0001)
Eastern Sub-Saharan Africa	3.289 (2.797 - 3.887)	1446.6 (1271.0 - 1646.2)	6.764 (5.782 - 7.943)	1445.9 (1270.7 - 1644.7)	-0.0030 (-0.0035 - -0.0024)
Southern Sub-Saharan Africa	0.848 (0.730 - 0.989)	1477.7 (1294.8 - 1679.8)	1.177 (1.029 - 1.345)	1475.0 (1292.4 - 1675.9)	-0.0070 (-0.0104 - -0.0036)
Western Sub-Saharan Africa	3.308 (2.816 - 3.913)	1451.4 (1273.6 - 1651.1)	7.590 (6.493 - 8.916)	1456.3 (1277.9 - 1656.2)	0.0137 (0.0101 - 0.0174)

ASR – Age-Standardized Rate, EAPC – Estimated Annual Percentage Change, UI – Uncertainty interval.

Table S2. The DALYs cases and ASR per 100,000 of urticaria in 1990 and 2019, and the EAPC of DALYs spanning 1990-2019

	1990		2019		1990-2019
	DALYs cases	ASR per 100,000	DALYs cases	ASR per 100,000	EAPC
	No. (95% UI)	No. (95% UI)	No. (95% UI)	No. (95% UI)	No. (95% UI)
Global	2889695.3 (1881485.9 - 4200997.5)	51.6 (33.8 - 74.2)	3898838.6 (2554225.4 - 5584365.6)	51.9 (34.0 - 75.1)	0.0338 (0.0287 - 0.0388)
Sex					
Male	1187604.9 (776134.6 - 1720442.3)	41.6 (27.3 - 60.1)	1613269.8 (1051942.8 - 2320511.4)	42.5 (27.8 - 61.7)	0.0878 (0.0823 - 0.0932)
Female	1702090.3 (1112433.9 - 2468057.8)	61.7 (40.5 - 88.3)	2285568.8 (1499168.1 - 3272442.3)	61.5 (40.4 - 88.8)	-0.0013 (-0.0061 - 0.0036)
Social-demographic index					
Low SDI	334010.1 (218227.5 - 489455.3)	53.5 (35.1 - 77.4)	678840.9 (443143.6 - 992715.1)	53.2 (34.9 - 76.6)	-0.0173 (-0.0196 - -0.0149)
Low-middle SDI	692177.7 (450787.7 - 1008459.3)	54.4 (35.6 - 78.5)	979311.1 (639278.3 - 1420447.6)	54.7 (35.8 - 79.1)	0.0281 (0.0243 - 0.0320)
Middle SDI	897567.0 (584515.9 - 1299659.9)	49.2 (32.1 - 70.7)	1149480.9 (752418.0 - 1643055.5)	50.2 (32.8 - 72.2)	0.0836 (0.0780 - 0.0892)
High-middle SDI	594152.8 (387212.6 - 856327.3)	52.2 (34.1 - 75.2)	656089.3 (432160.1 - 936970.6)	51.2 (33.5 - 73.7)	-0.0470 (-0.0638 - -0.0301)
High SDI	370391.8 (244053.2 - 522773.6)	47.5 (31.2 - 67.4)	433027.6 (285343.5 - 609777.2)	47.5 (31.3 - 67.6)	-0.0004 (-0.0103 - 0.0095)
Region					
Central Asia	51231.8 (33493.6 - 74614.6)	66.3 (43.6 - 95.8)	62416.7 (40782.9 - 90250.0)	66.2 (43.3 - 96.0)	-0.0050 (-0.0063 - -0.0036)
Central Europe	81794.8 (53933.2 - 118171.5)	72.3 (47.5 - 104.2)	67169.3 (44596.1 - 95460.2)	71.9 (47.2 - 103.4)	-0.0034 (-0.0084 - 0.0017)
Eastern Europe	146607.4 (95797.1 - 211523.6)	70.0 (45.7 - 102.1)	125843.9 (82383.2 - 180930.6)	70.1 (46.0 - 102.1)	0.0068 (0.0050 - 0.0085)
Australasia	10667.4 (6964.2 - 15115.8)	54.3 (35.7 - 77.5)	14694.8 (9608.9 - 21046.6)	54.5 (35.3 - 78.2)	0.0110 (0.0076 - 0.0144)
High-income Asia Pacific	76764.3 (50531.2 - 110521.8)	47.7 (31.3 - 69.0)	74570.0 (49254.8 - 105635.9)	47.4 (31.1 - 68.4)	-0.0149 (-0.0172 - -0.0126)
High-income North America	139663.5 (92687.9 - 197132.9)	53.0 (35.1 - 74.9)	171109.0 (114326.9 - 239859.8)	53.5 (35.5 - 75.6)	0.0018 (-0.0116 - 0.0153)
Southern Latin America	23038.1 (15073.3 - 33464.0)	45.9 (29.9 - 66.5)	28928.2 (18987.6 - 41298.2)	45.8 (30.0 - 66.2)	0.0001 (-0.0024 - 0.0026)

Western Europe	136808.9 (90836.4 - 191363.0)	34.8 (22.9 - 49.1)	155143.4 (103509.7 - 217649.1)	34.9 (23.0 - 49.4)	0.0161 (0.0135 - 0.0187)
Andean Latin America	20290.1 (13291.1 - 29655.9)	48.0 (31.5 - 69.2)	30723.5 (19985.1 - 44502.2)	48.1 (31.4 - 69.8)	0.0078 (0.0055 - 0.0101)
Caribbean	17706.5 (11547.5 - 25817.9)	48.1 (31.5 - 69.8)	21826.7 (14363.9 - 31358.7)	48.0 (31.5 - 69.1)	-0.0069 (-0.0084 - -0.0054)
Central Latin America	89957.4 (58494.7 - 131864.7)	49.6 (32.3 - 72.0)	121438.7 (79078.5 - 175939.7)	49.7 (32.4 - 72.3)	0.0000 (-0.0013 - 0.0013)
Tropical Latin America	82324.2 (53547.9 - 119971.2)	50.6 (33.2 - 73.3)	106852.1 (69841.2 - 154007.6)	50.8 (33.1 - 73.5)	0.0073 (0.0052 - 0.0093)
North Africa and Middle East	220985.6 (144152.2 - 323560.9)	56.9 (37.1 - 82.0)	350946.2 (229607.5 - 508231.2)	57.0 (37.4 - 82.8)	0.0094 (0.0077 - 0.0111)
South Asia	726277.0 (473731.7 - 1055685.6)	58.3 (38.3 - 83.8)	1068940.9 (696805.2 - 1546075.7)	59.1 (38.6 - 85.3)	0.0518 (0.0497 - 0.0540)
East Asia	561250.0 (364288.0 - 810674.3)	44.6 (29.2 - 64.7)	595886.5 (392144.5 - 845623.1)	44.6 (29.1 - 64.8)	0.0022 (0.0006 - 0.0039)
Oceania	2934.5 (1910.8 - 4278.0)	41.5 (27.2 - 59.3)	5853.3 (3843.8 - 8629.9)	41.4 (27.1 - 59.8)	0.0002 (-0.0018 - 0.0023)
Southeast Asia	220182.8 (145247.1 - 315169.9)	44.3 (29.3 - 63.4)	297242.9 (196270.5 - 423283.2)	44.6 (29.4 - 63.7)	0.0254 (0.0236 - 0.0271)
Central Sub-Saharan Africa	31361.3 (20644.9 - 46005.1)	47.5 (31.4 - 68.7)	71958.9 (46757.3 - 105267.1)	47.8 (31.5 - 68.9)	0.0219 (0.0178 - 0.0261)
Eastern Sub-Saharan Africa	110306.9 (71728.9 - 162984.1)	48.8 (31.8 - 70.8)	230139.9 (150650.3 - 338951.0)	49.0 (32.1 - 71.1)	0.0238 (0.0208 - 0.0269)
Southern Sub-Saharan Africa	28938.1 (18881.3 - 42307.8)	50.1 (32.8 - 72.7)	40035.0 (25993.7 - 58297.4)	50.0 (32.6 - 72.5)	-0.0101 (-0.0115 - -0.0088)
Western Sub-Saharan Africa	110604.6 (72061.7 - 162297.9)	48.9 (32.0 - 70.8)	257118.5 (168026.7 - 376269.7)	49.3 (32.3 - 71.5)	0.0310 (0.0247 - 0.0373)

ASR – Age-Standardized Rate, EAPC – Estimated Annual Percentage Change, UI – Uncertainty interval.

Figure S1. The urticaria global ASR (per 10^5) prevalence map and EAPC of prevalence map in 2019 by countries and territories. **Panel A.** ASR (per 10^5) prevalence map. **Panel B.** EAPC of prevalence map.

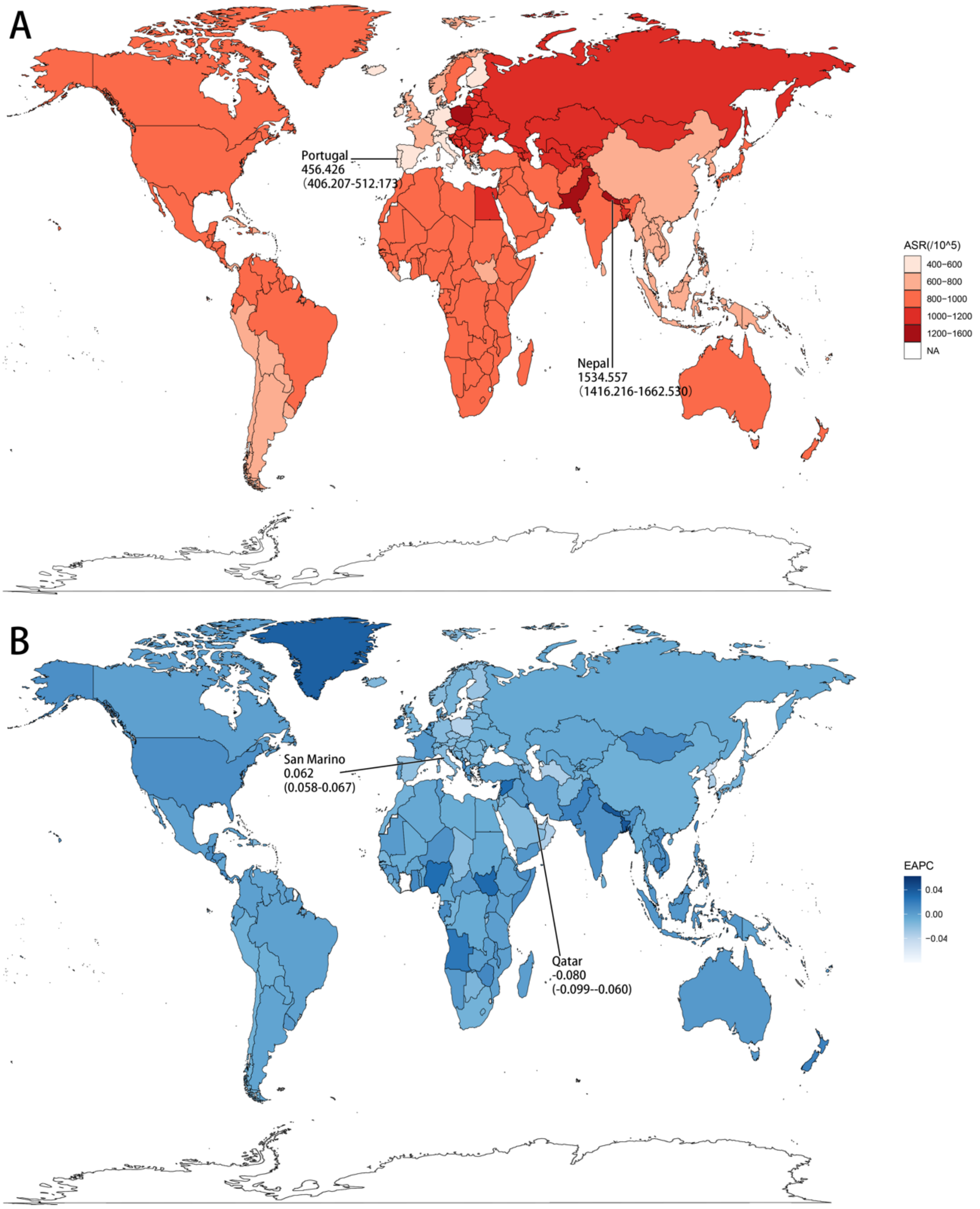

Figure S2. The urticaria global ASR (per 10^5) DALYs map and EAPC of DALYs map in 2019 by countries and territories. **Panel A.** ASR (per 10^5) DALYs map. **Panel B.** EAPC of DALYs map.

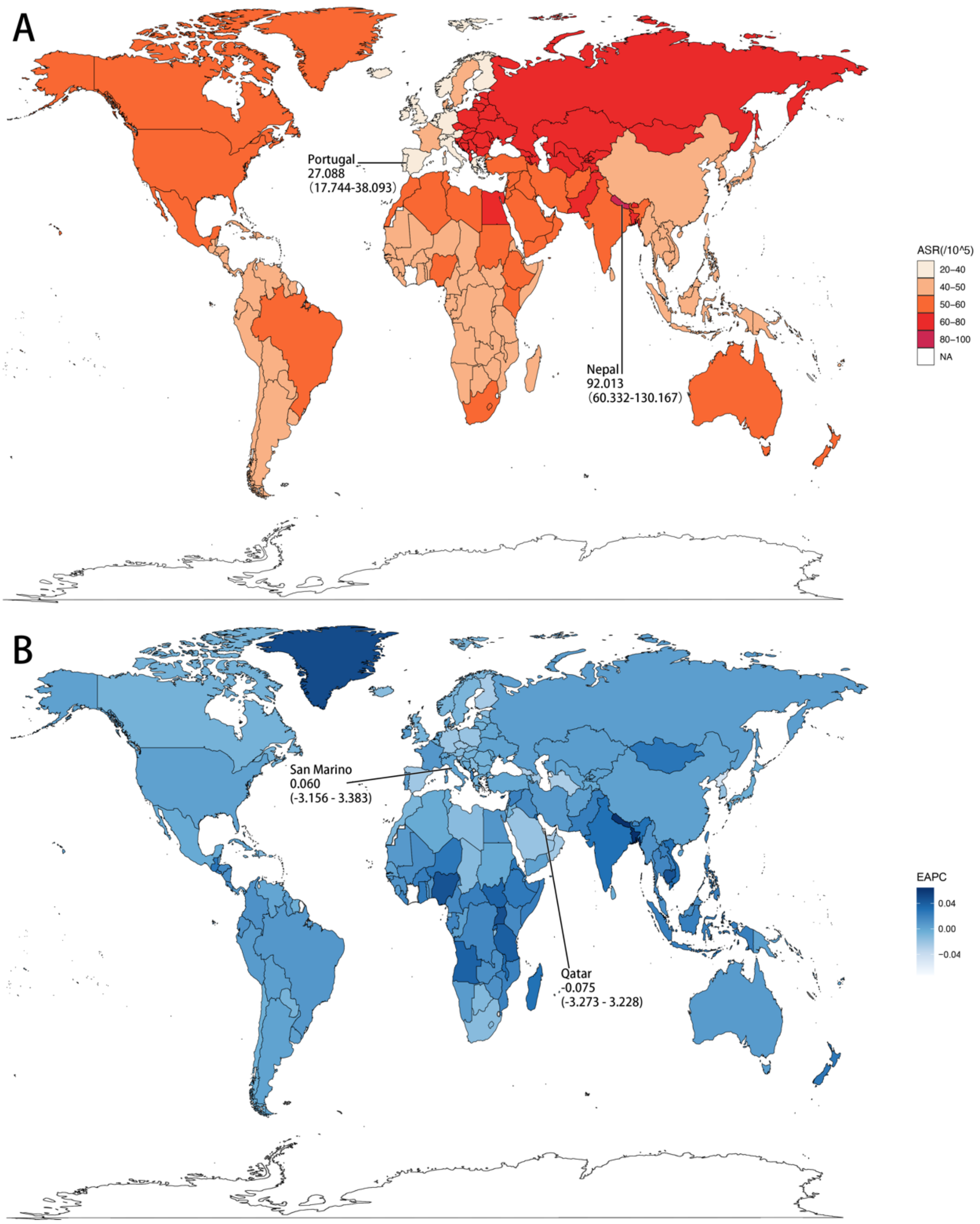

Figure S3. The prevalence cases and prevalence rates (per 100,000 population) of urticaria among age and gender in 2019. **Panel A.** Prevalence cases. **Panel B.** Prevalence rates.

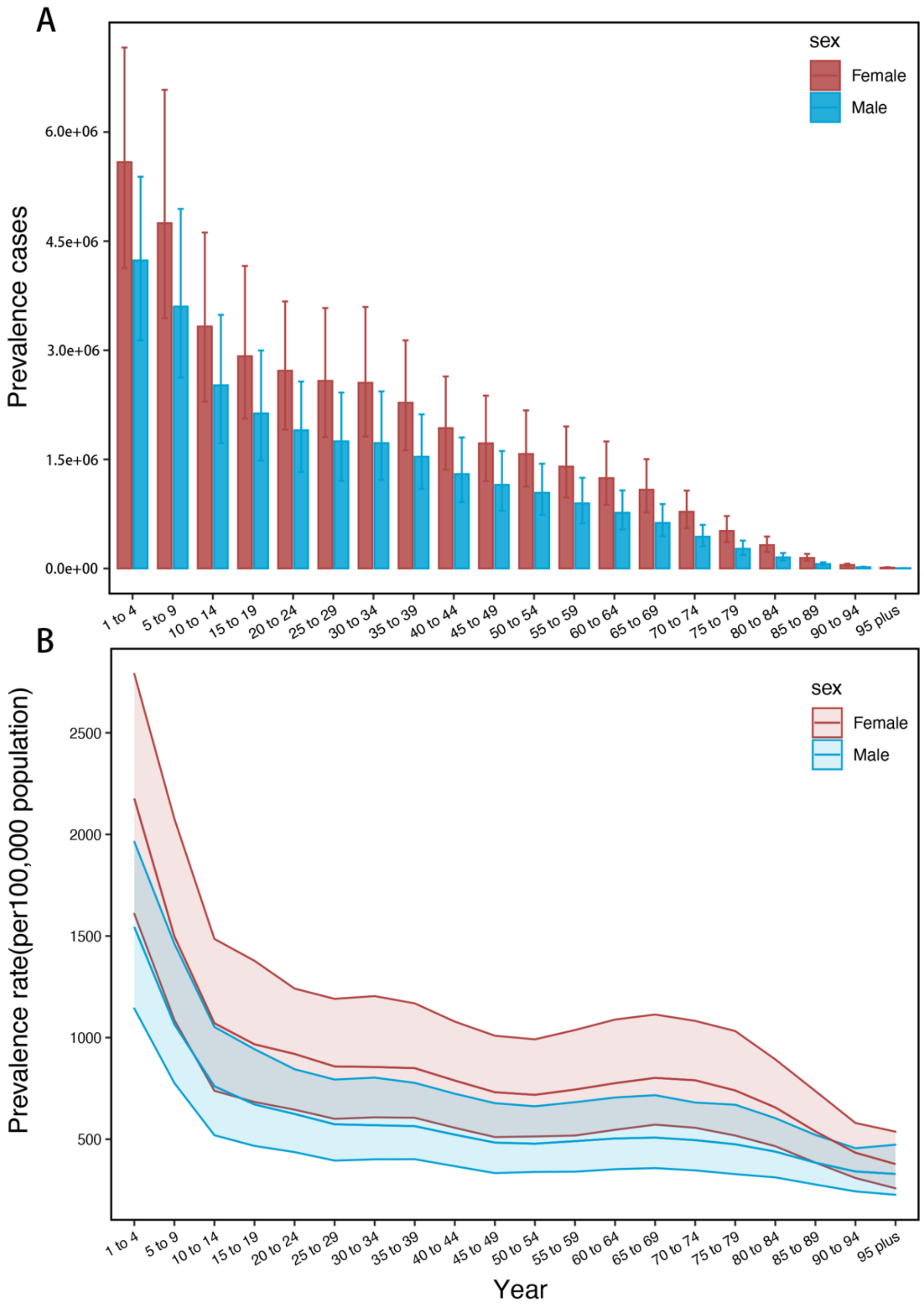

Figure S4. The incidence cases and incidence rates (per100,000 population) of urticaria among age and gender in 2019. **Panel A.** Incidence cases. **Panel B.** Incidence rates.

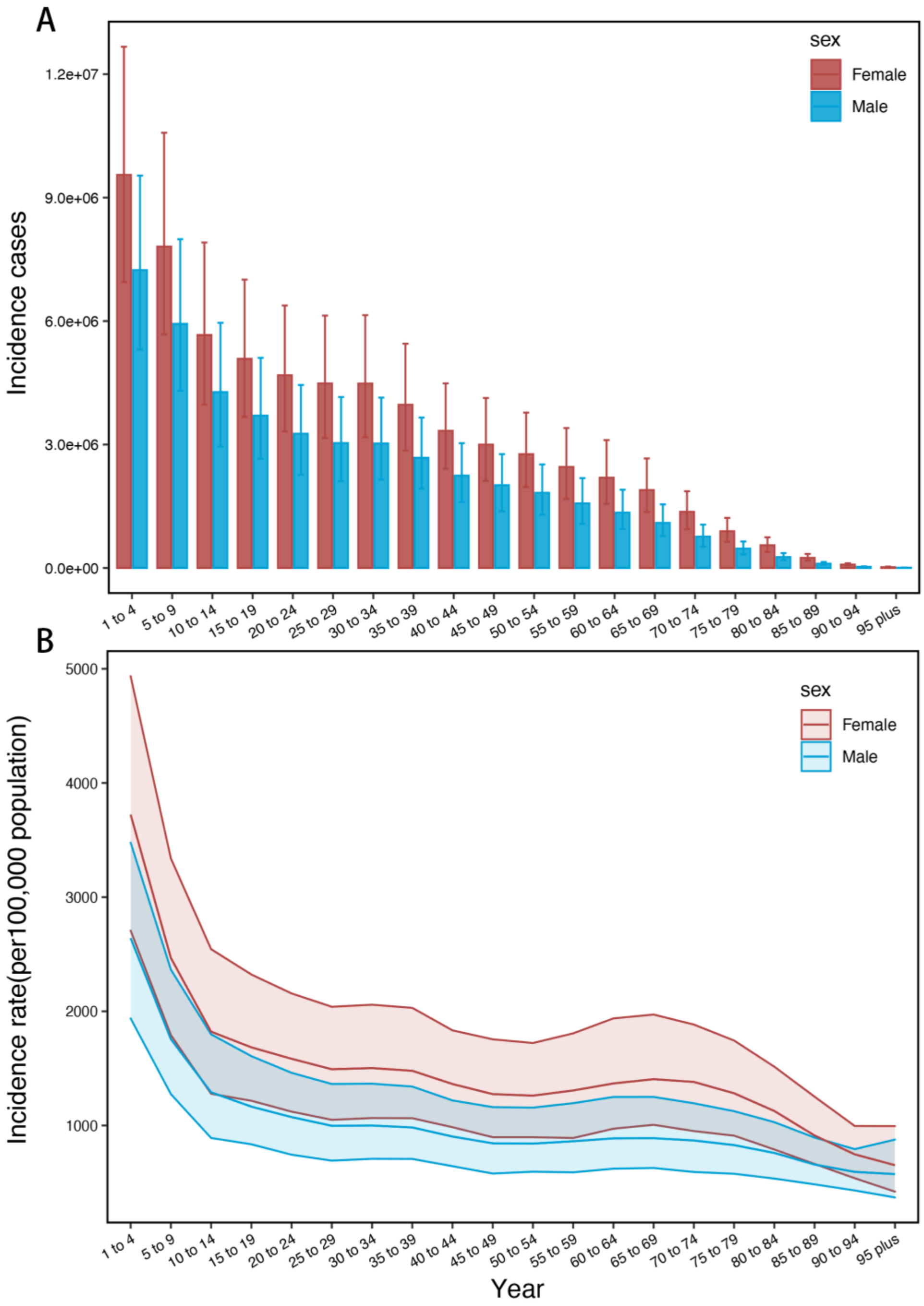

Figure S5. The DALYs cases and DALYs rates (per100,000 population) of urticaria among age and gender in 2019. **Panel A.** DALYs cases. **Panel B.** DALYs rates.

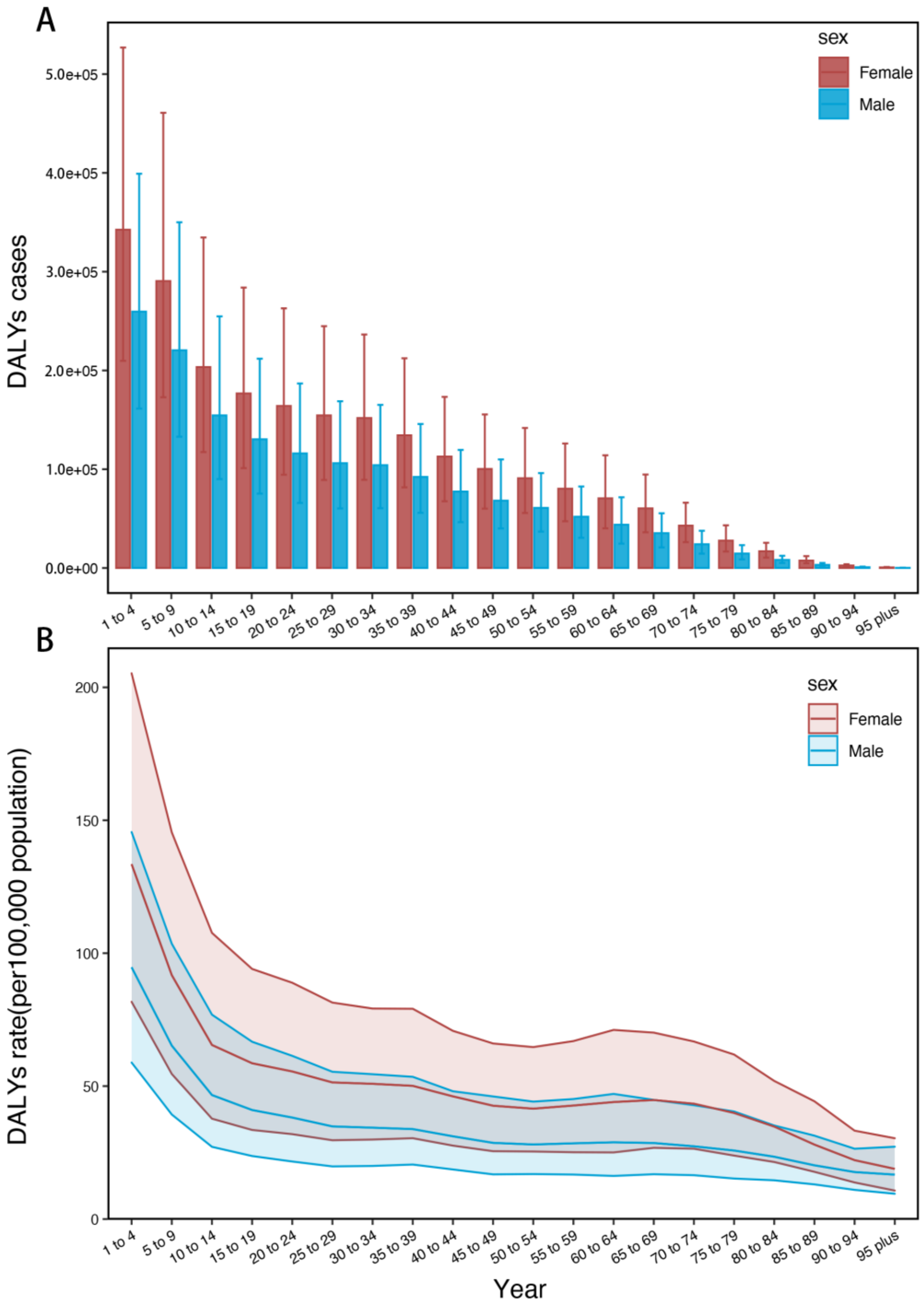

Figure S6. The correlation between urticaria ASIR and SDI levels. **Panel A.** Regional ASIR and SDI correlation from 1990 to 2019. **Panel B.** National ASIR and SDI correlation in 2019.

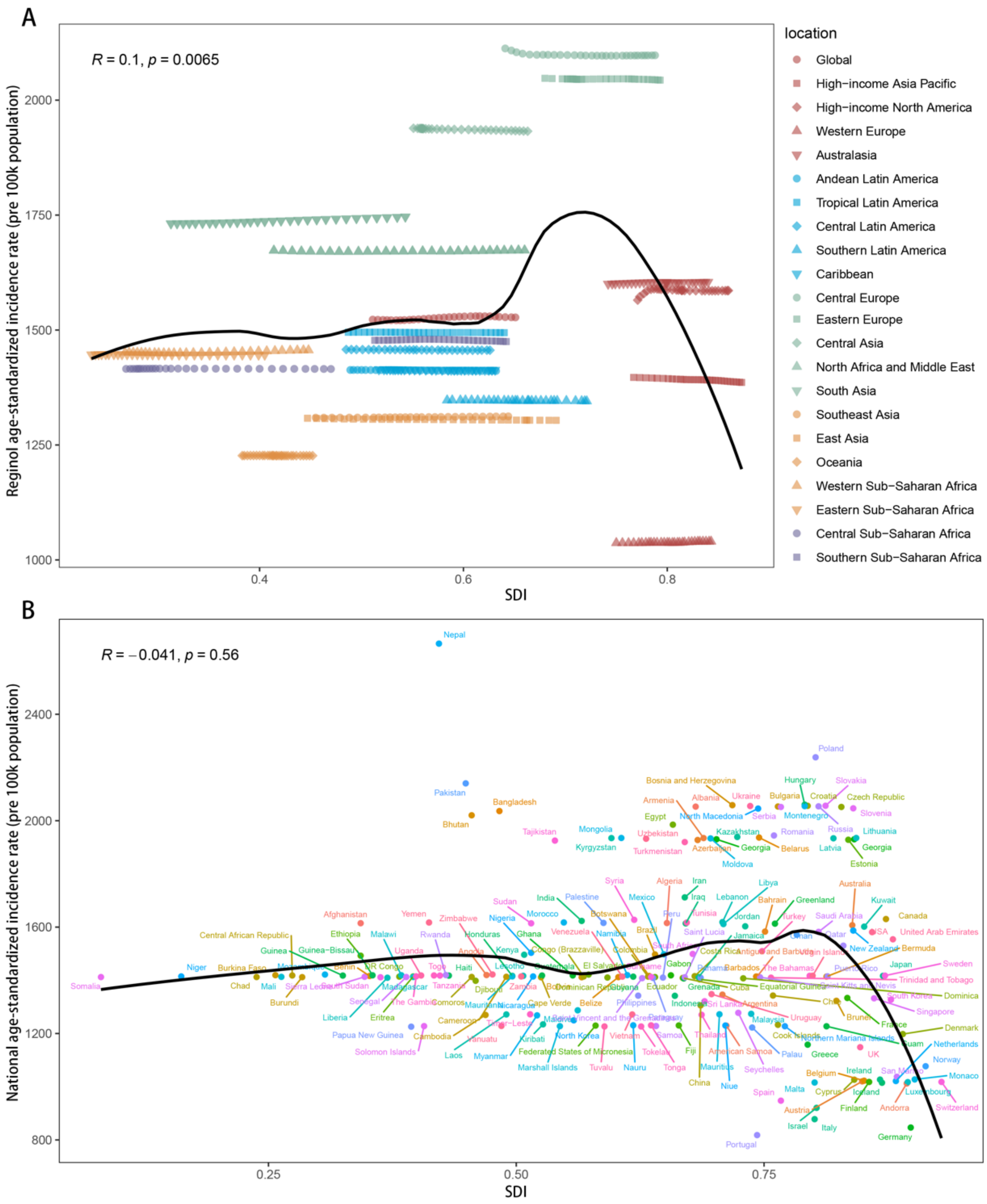

Figure S7. The correlation between urticaria age-standardized DALYs and SDI levels. **Panel A.** Regional age-standardized DALYs and SDI correlation from 1990 to 2019. **Panel B.** National age-standardized DALYs and SDI correlation in 2019.

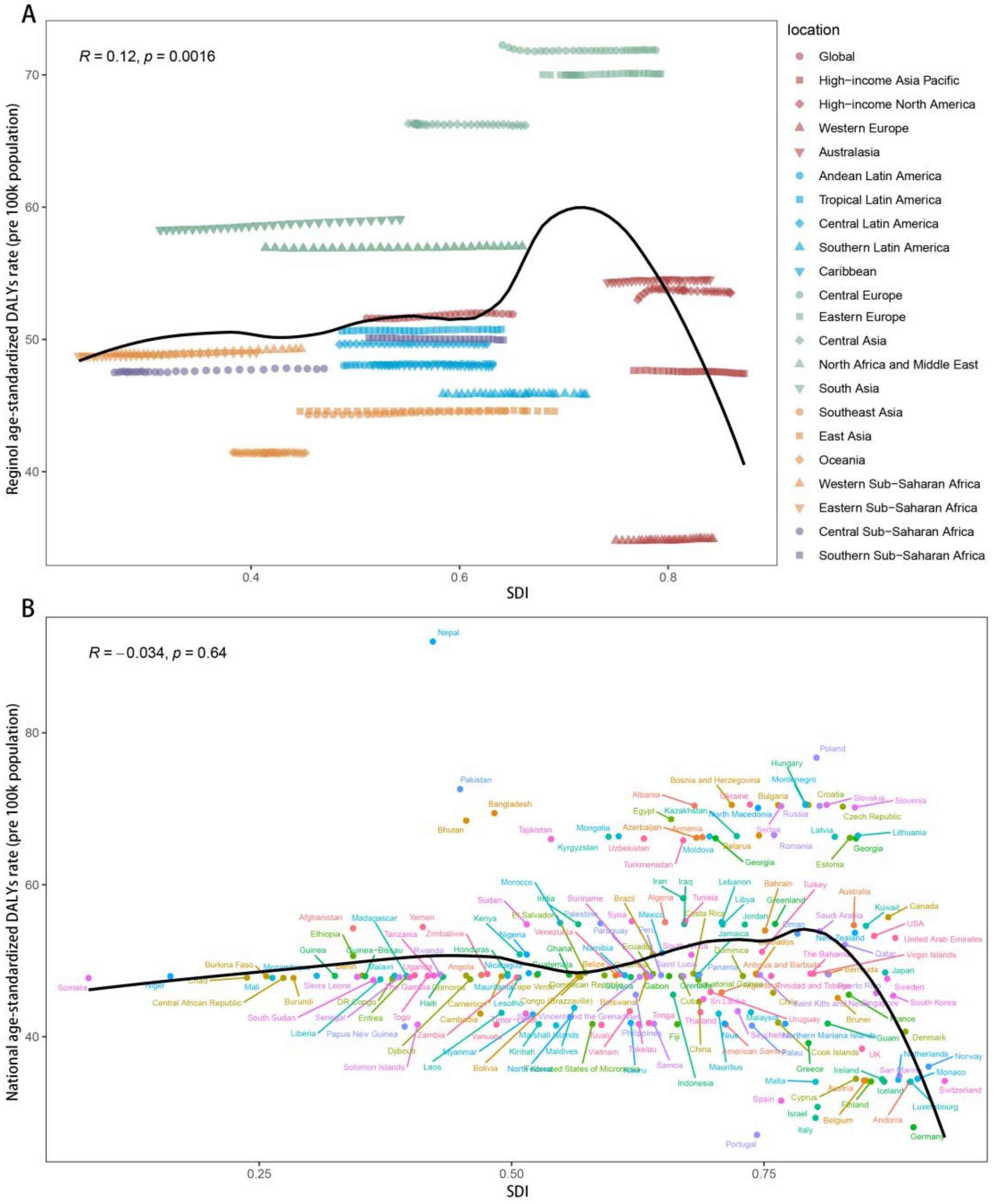

Figure S8. The urticaria incidence cases of 5 age group in Global and 5SDI level from 1990 to 2019.

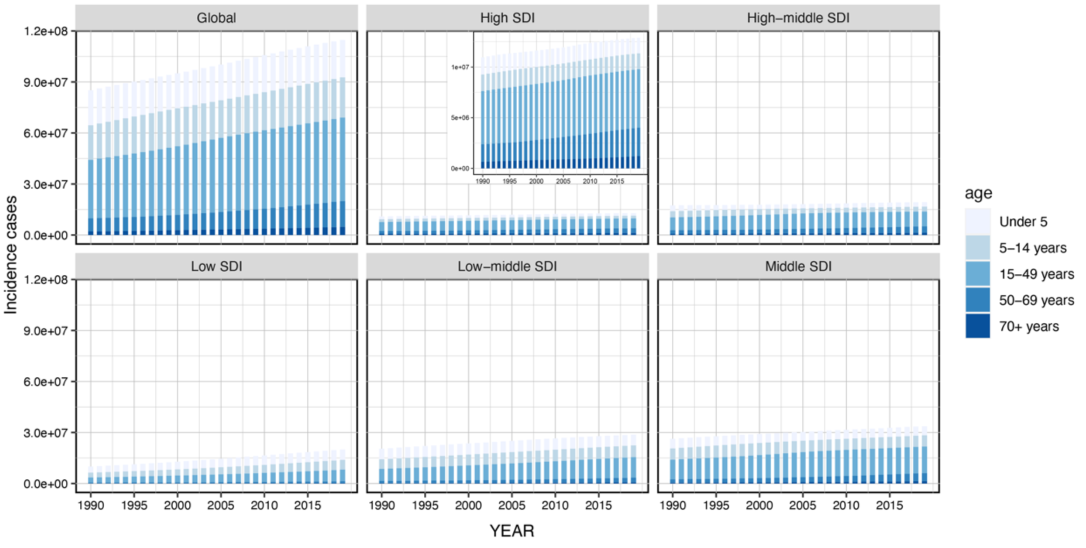

Figure S9. The urticaria DALYs cases of 5 age group in Global and 5SDI level from 1990 to 2019.

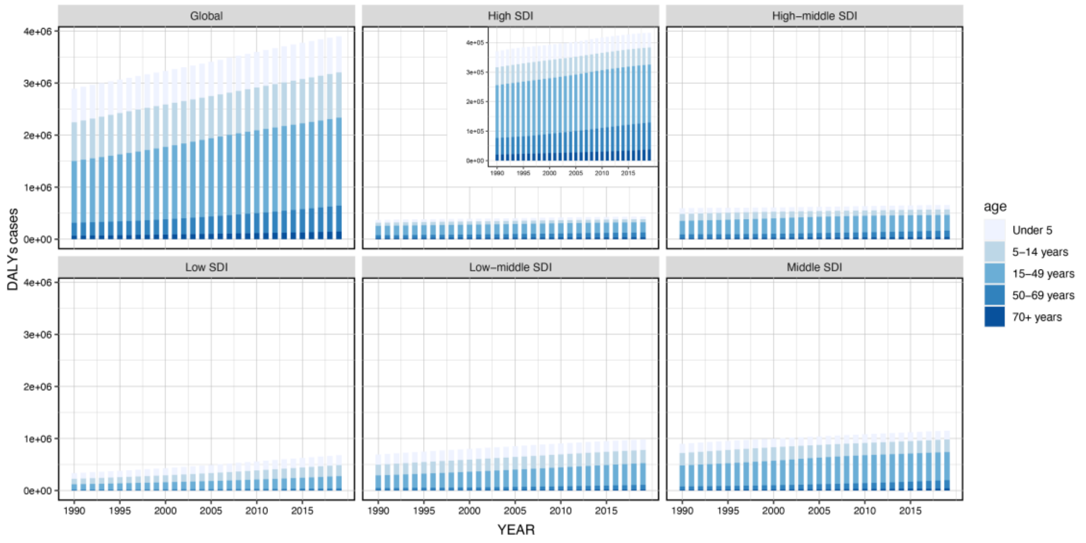

Figure S10. Temporal trends of ASPR in 5SDI levels by Jointpoint regression model. **Panel A.** High-middle SDI. **Panel B.** Low-middle SDI. **Panel C.** High SDI. **Panel D.** Middle SDI. **Panel E.** Low SDI.

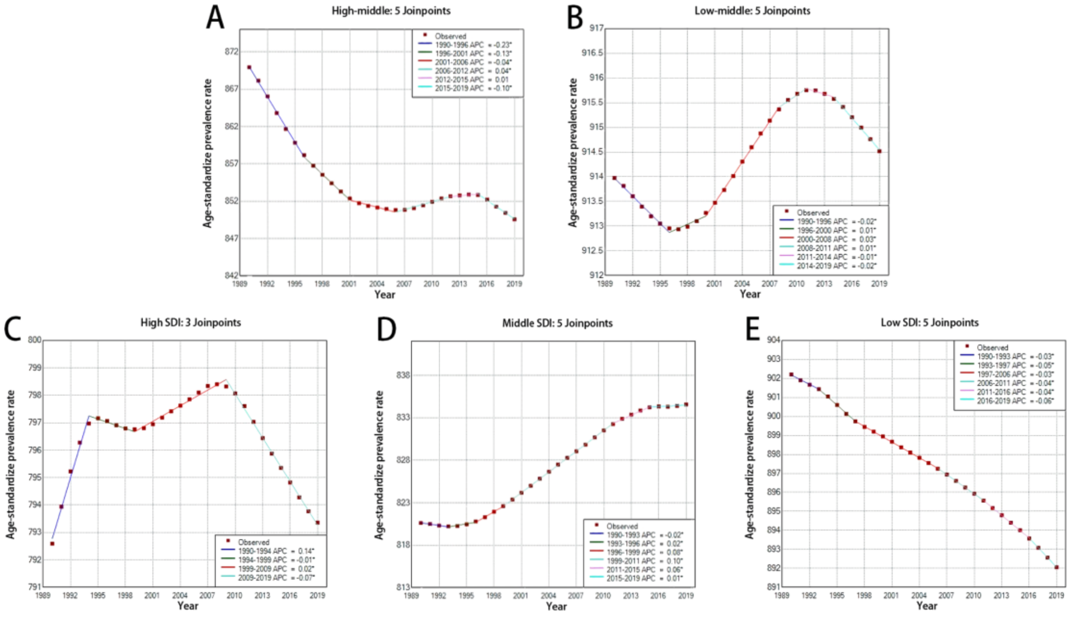

Figure S11. Temporal trends of ASIR in 5SDI levels by Jointpoint regression model. **Panel A.** High-middle SDI. **Panel B.** Low-middle SDI. **Panel C.** High SDI. **Panel D.** Middle SDI. **Panel E.** Low SDI.

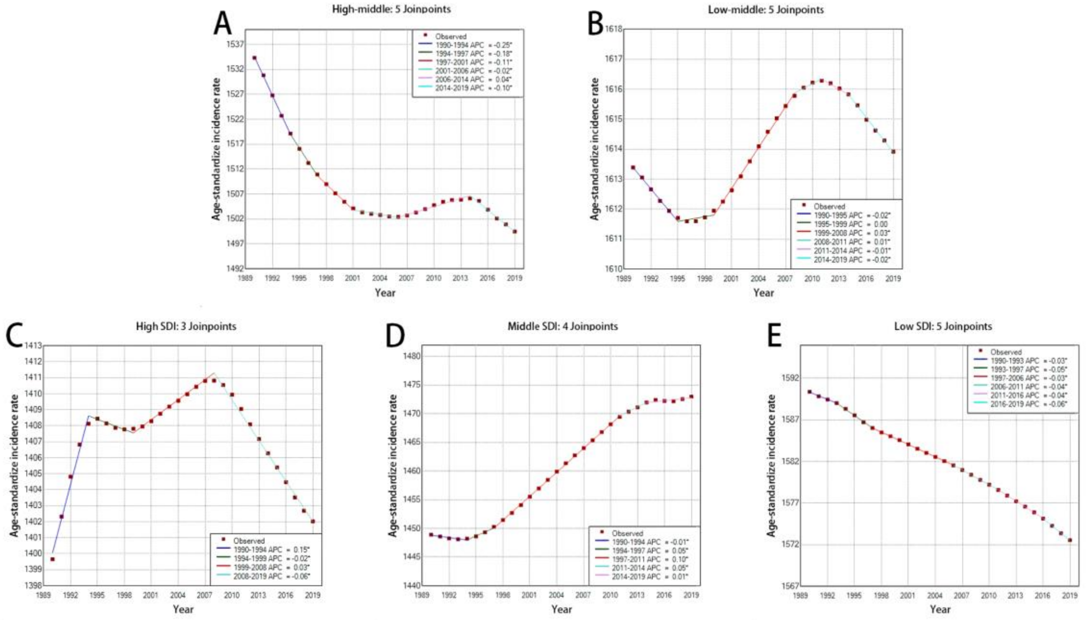

Figure S12. Temporal trends of age-standardized DALYs in 5SDI levels by Jointpoint regression model. **Panel A.** High-middle SDI. **Panel B.** Low-middle SDI. **Panel C.** High SDI. **Panel D.** Middle SDI. **Panel E.** Low SDI.

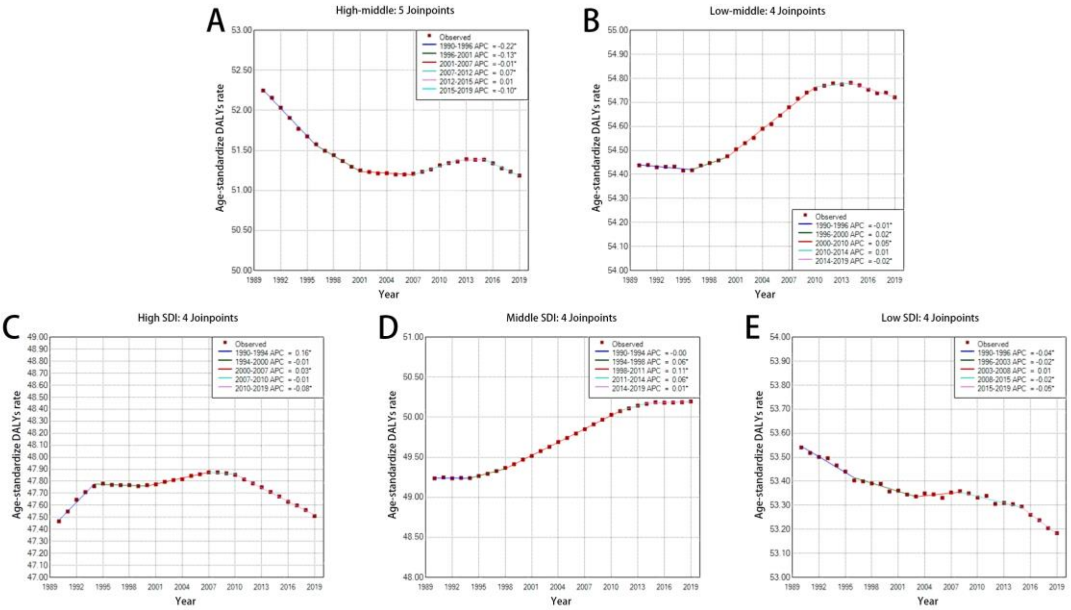

Supplement: Online Supplementary Document [file jogh-14-04095-s001.pdf]
